# Efficacy of Photobiomodulation in the Treatment of Cancer Chemotherapy-Induced Oral Mucositis: A Meta-Analysis with Trial Sequential Analysis

**DOI:** 10.3390/ijerph18147418

**Published:** 2021-07-12

**Authors:** Ali Hatem Manfi Al-Rudayni, Divya Gopinath, Mari Kannan Maharajan, Sajesh K. Veettil, Rohit Kunnath Menon

**Affiliations:** 1 School of Postgraduate Studies, International Medical University, Kuala Lumpur 57000, Malaysia; alihatem.manfiI@student.imu.edu.my; 2 Department of Oral Diagnostics & Surgical Sciences, International Medical University, Kuala Lumpur 57000, Malaysia; 3 Department of Pharmacy Practice, School of Pharmacy, International Medical University, Kuala Lumpur 57000, Malaysia; marikannan@imu.edu.my; 4 Department of Pharmacotherapy, College of Pharmacy, University of Utah, Salt Lake City, UT 84112, USA; Sajesh.Veettil@pharm.utah.edu; 5 Division of Restorative Dentistry, International Medical University, Kuala Lumpur 57000, Malaysia

**Keywords:** Photobiomodulation, low-level laser, chemotherapy-induced oral mucositis, RCTs, trial sequential analysis, meta-analysis

## Abstract

Oral mucositis is a debilitating complication of chemotherapy, characterized by erythema, ulcers and oedema of the oral mucosa. This review aimed to evaluate the efficacy of Photobiomodulation in the treatment of oral mucositis using meta-analysis and trial sequential analysis, and also to assess the quality of the results by Grading of Recommendations, Assessment, Development and Evaluation (GRADE). A comprehensive search of three databases, including Embase, Medline and Central, was performed to identify randomized controlled trials studying the efficacy of Photobiomodulation in the treatment of cancer chemotherapy-induced oral mucositis. The primary outcome was reduction in the severity of oral mucositis. Secondary outcomes were pain relief, duration of oral mucositis and adverse effects. The meta-analysis was performed using the random-effects model, and random errors of the meta-analyses were detected by trial sequential analysis. A total of 6 randomized controlled trials with 398 participants were included in our analysis. Photobiomodulation significantly reduced the severity of oral mucositis when compared to sham radiation (RR 0.43, 95% CI 0.20 to 0.93; *p* < 0.05). Sensitivity analysis by excluding trials with high risk of bias reiterated the robustness of our results (RR 0.28, 95% CI 0.16 to 0.48). Trial sequential analysis illustrated that the evidence from the meta-analysis was conclusive. The result of the meta-analyses with trial sequential analysis illustrated that Photobiomodulation is an effective therapeutic intervention for the treatment of oral mucositis, and the evidence gathered can be considered conclusive with a moderate level of certainty according to GRADE. Further trials are recommended to standardize the laser parameters required for the optimal effect.

## 1. Introduction

Supportive care plays an important role in improving cancer patient survival by enhancing patient adherence and reducing treatment interruptions and treatment-related mortality [1]. Oral mucositis (OM) is an inflammation affecting the oral mucosa, characterized by erythematic, ulcerative painful lesions affecting the lining mucosa of the oral cavity. Patients with OM usually present with a burning sensation and pain in the mouth, ulcers, sometimes hemorrhage, dysphagia and dysarthria [2,3]. OM severity is evaluated using the World Health Organization oral toxicity scale (WHO-OTS) and National Cancer Institution Common Toxicity Criteria for Adverse Events (NCI-CTCAE) [4,5]. For many years, OM management relied almost exclusively on empirical therapies. The Multinational Association for Supportive Care in Cancer (MASCC/ISOO) guidelines recommended against the use of certain empirical therapies as some of them were shown to be more harmful than effective, such as antibiotics [6,7]. In the medical literature, there are a plethora of interventions that were introduced as a potential treatment for OM [5]. However, only a few agents were shown to be effective, including Photobiomodulation (PBM), which has shown promising effectiveness in the management of OM in recent years [7,8].

PBM is characterized by irradiating the oral mucosa with a low-energy laser. PBM can stimulate tissue regeneration, reduce inflammation and control pain [9,10]. The light used is of low intensity when compared to other types of laser therapy that are utilized in surgical interventions, such as lasers for ablation, cutting and coagulation [11]. PBM stimulates the production of growth factors and the proliferation of keratinocytes, leading to minimization of mucosal damage and accelerating the wound healing process [12].

PBM has been previously proven to be effective for the prevention of OM by well-designed trials, and the MASCC/ISOO guidelines have recommended the use of PBM for the prevention of OM induced by chemotherapy and radiotherapy, supported by a high level of evidence [7,8,13,14]. The guidelines recommended the use of PBM for the prevention of OM in patients receiving high-dose chemotherapy conditioning for Hematopoietic Stem Cell Transplantation (HSCT), and Head and Neck Cancer (HNC) patients receiving radiotherapy with or without chemotherapy [7,8]. The MASCC/ISOO guidelines lack any recommendations or suggestions regarding the use of PBM as a treatment modality for OM. Nevertheless, multiple well-designed RCTs with a low risk of bias have emerged in the previous years, investigating the efficacy of PBM in the treatment of OM. The objective of the current systematic review with meta-analysis and trial sequential analysis (TSA) was to provide reliable estimates on the efficacy and safety of PBM to facilitate evidence-based decision-making on its use as a treatment modality for OM in patients undergoing cancer chemotherapy.

## 2. Materials and Methods

### 2.1. Study Design

A systematic review and meta-analysis of the efficacy and safety of PBM in the treatment of OM in patients undergoing cancer treatment was performed according to the general principles of the Cochrane Handbook for Systematic Reviews of Interventions, and reported according to the Preferred Reporting Items for Systematic Reviews and Meta-Analyses (PRISMA) extension statement. [15].

The protocol for the systematic review was registered in the international prospective register of systematic reviews (PROSPERO ID: CRD42020159741).

### 2.2. Data Resources and Search Strategy

Relevant studies were identified through a systematic search of Medline, Embase, and Cochrane Central Register of Controlled Trials from inception until 07 June 2020 by using subject headings and free-text terms. In addition, published systematic reviews were searched for additional studies. Two sets of search terms were combined: terms for OM in patients with cancer treatment and terms for PBM. The search strategy was developed in Medline and then applied to other databases. A detailed description of the search strategy is provided in Supplementary Table S1.

### 2.3. Study Selection

#### Inclusion Criteria

Studies included in the systematic review were randomized controlled trials (RCTs) that met the following inclusion criteria:

P: Patients who developed OM after chemotherapy.

I: Intervention was any type of PBM at any dose.

C: Control groups received sham radiation, no treatment, or another active intervention.

O: primary Outcome was the severity of OM. Secondary outcomes were the duration of OM, pain relief and adverse effects.

### 2.4. Data Extraction and Quality Assessment

Titles and abstracts were screened independently for eligible studies (by A.H.M. and R.K.M.), followed by full-text reading. Ineligible studies were excluded and the reason for exclusion was documented in Supplementary Table S2. Data were extracted independently and in duplicate by two reviewers (A.H.M. and R.K.M.), into a data extraction form. The form was created in accordance with the Cochrane handbook for systematic reviews of intervention guidelines [15]. The data from RCTs were separated into the following sections, namely, study characteristics, population characteristics, intervention characteristics, outcome definitions and measures. For all outcomes, the initial number of participants randomized to each trial arm was used in the analysis regardless of how the authors of the original trials had analyzed the data, referred to as Intention to Treat (ITT) analysis. For risk of bias assessment, two reviewers assessed the risk of bias independently (A.H.M. and R.K.M.) using the revised Cochrane risk of bias tool (ROB 2.0) [16,17].

### 2.5. Data Synthesis

The meta-analysis was performed with the DerSimonian and Laird random-effects model to estimate pooled risk ratios and 95% confidence intervals, incorporating heterogeneity within and between studies [18], with Stata version 15.0 (StataCorp, College Station, TX, USA). If a direct comparison was based on two or more studies, heterogeneity between trials was assessed by considering the I^2^ statistics, where an I^2^ estimate ≥ 50% was interpreted as evidence of substantial levels of heterogeneity [19]. Publication bias and small study effects were assessed using funnel plot asymmetry testing and Egger’s regression test, respectively [20]. A sensitivity analysis was carried out by excluding trials at high risk of bias to assess the robustness of the meta-analysis. Subgroup analyses were carried out based on the type of malignancy and cancer treatment protocol.

The risks of random errors were assessed by performing the trial sequential analysis (TSA) using the TSA software package (available at http://www.ctu.dk) (accessed on 18/01/2021), which combines information size estimation for meta-analysis (cumulated sample size of included trials) with an adjusted threshold for statistical significance in the cumulative meta-analysis [21]. TSA provides the necessary sample size for the meta-analysis and boundaries that determine whether the evidence in our meta-analyses is reliable and conclusive [21]. The Grading of Recommendation, Assessment, Development and Evaluation (GRADE) approach was used to rate the quality of evidence (high, moderate, low and very low) [22].

## 3. Results

### 3.1. Study Selection

**Figure 1:**
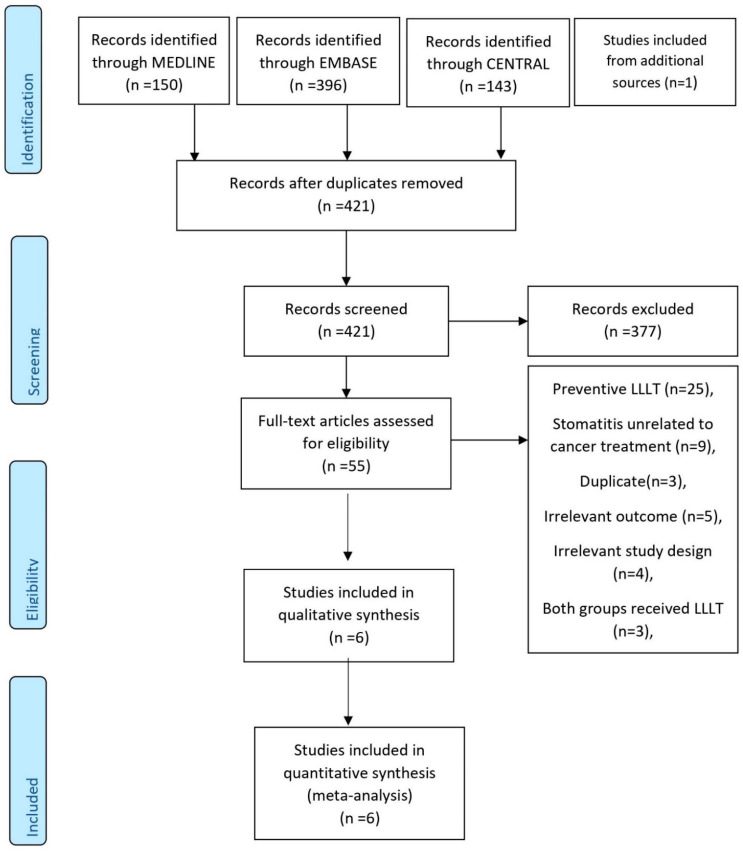
PRISMA flow chart for the included studies. RCT: randomized control trial, CT: chemotherapy, RT: radiotherapy, GaAIAs: gallium aluminum arsenide, He Ne; helium-neon, low-energy laser.

The PRISMA flow chart demonstrates the selection process of the included studies, as illustrated in Figure 1. A total of 690 articles were identified after the initial search and 421 were selected after the removal of duplicates. After the title and abstract screening, 377 studies were removed which did not fit the inclusion criteria. Out of the remaining 55 studies, only 6 were selected as the other 49 were excluded because of specific reasons, as outlined in Supplementary Table S2.

### 3.2. Characteristics of the Selected Studies

The characteristics of the included studies are provided in Table 1. A total of 6 trials [23,24,25,26,27,28] were included in the final meta-analysis, encompassing 398 participants. Two trials were conducted in Brazil [25,26], two in Italy [23,24] and one each in France [27] and Belgium [28]. With respect to the laser source, a diode laser was used in two trials [23,24], GaAIAs in two trials [25,26], He-Ne in one trial [27] and the LEL (low-energy laser) with a scanning laser combined with an infra-red laser in one trial [28]. The dose ranged from 2 J/cm^2^ to 6 J/cm^2^. Evaluation of OM was performed by The World Health Organization criteria in three studies [23,24,27], the National Cancer Institute (version 2.0) in two studies [25,26] and the European Organization for Research and Treatment of Cancer Scale in one study [28].

### 3.3. Risk of Bias

**Figure 2:**
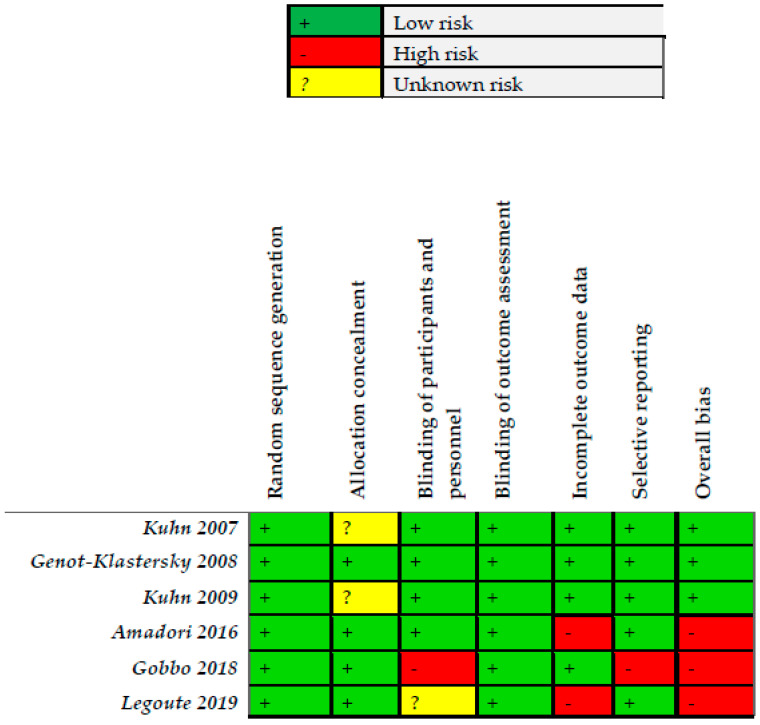
Risk of bias of the included trails for PBM.

Three studies were graded as low risk of bias [25,26,28] and three were graded as high risk of bias [23,24,27] (Figure 2).

### 3.4. Reduction in the Severity of OM by PBM

**Figure 3:**
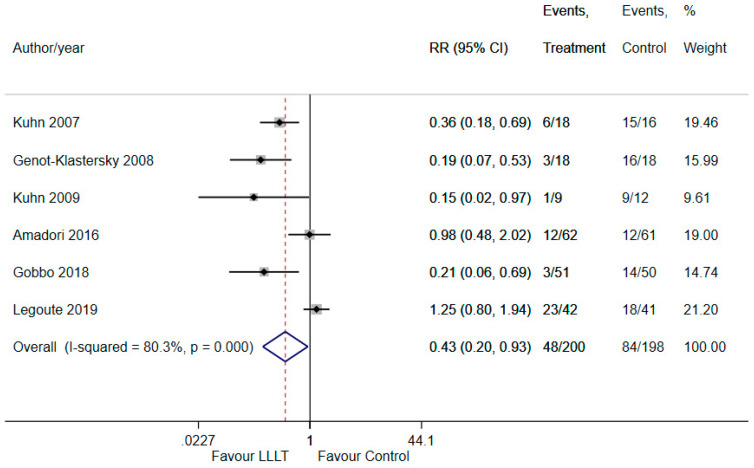
The forest plot illustrating the therapeutic benefit of PBM in reducing the severity of OM.

PBM significantly reduced the risk of severe OM when compared to sham irradiation (RR 0.43, 95% CI 0.20 to 0.93; *p* < 0.05) (Figure 3). A substantial level of heterogeneity was identified (I^2^ = 80.3%). The funnel plot and eggers test indicated publication bias (Supplementary Figure S1).

### 3.5. Sensitivity Analysis

**Figure 4:**
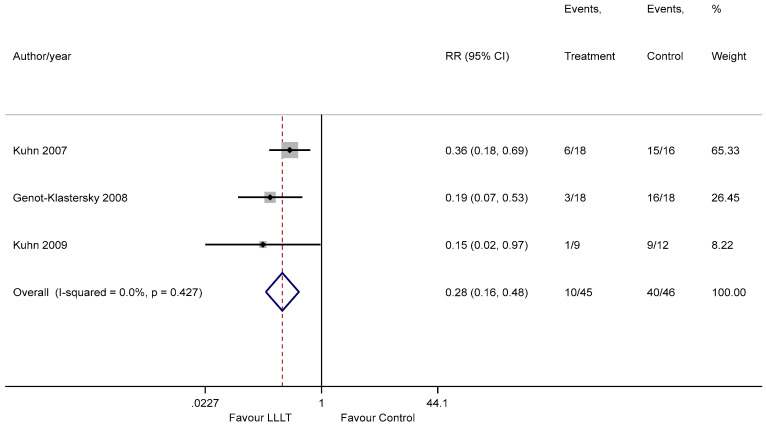
The sensitivity analysis forest plot illustrating the pooled estimate of PBM in reducing the severity of OM.

We carried out a sensitivity analysis by excluding the three trials with high ROB. The same effect was demonstrated pertaining to the efficacy of PBM for the reduction in severity of OM, with a RR of 0.28 (95% CI 0.16 to 0.48) (Figure 4); however, heterogeneity was reduced to 0%, depicting high-quality evidence.

### 3.6. Trial Sequential Analysis

**Figure 5:**
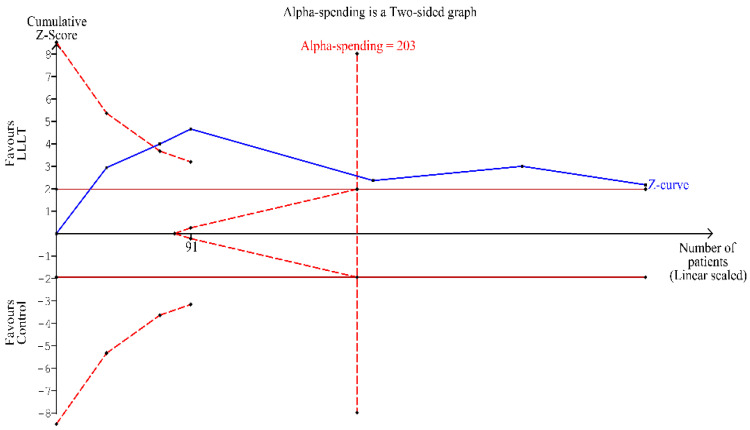
Trial sequential analysis evaluating the therapeutic benefit of PBM in inducing partial clinical resolution of OM using random-effects meta-analysis.

TSA comparing PBM to sham irradiation for the treatment of OM was undertaken with a type 1 error of 5% and a type II error of 20% by using a random-effects model. The information size (*n* = 203) was calculated using an anticipated intervention effect of RR = 0.43 (Figure 3) and a control event proportion of 86.9% based on low ROB trials. The Z-curve (blue line) crossed the conventional (cumulative Z-score between −2 and +2) boundary, indicating a significant benefit of the intervention, as demonstrated in the meta-analysis (Figure 5). The number of patients included in the meta-analysis (*n* = 398) exceeded the required information size. Moreover, the cumulative Z-curve also crossed the alpha-spending boundary (red-dotted line). Hence, the evidence obtained from the meta-analysis can be considered conclusive.

### 3.7. Duration of OM

**Figure 6:**
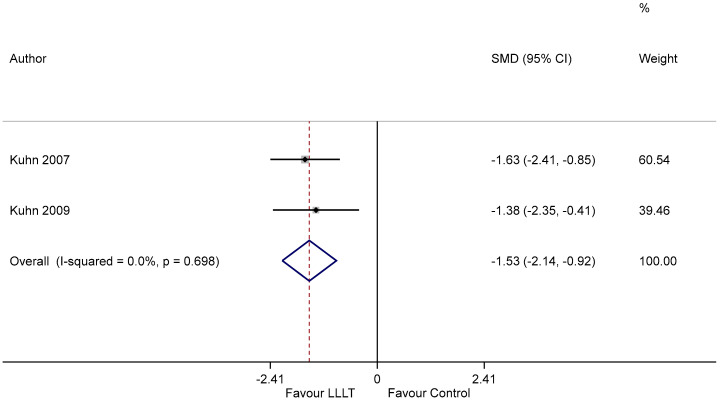
Forest plot illustrating the efficacy of PBM in reducing OM duration.

Two studies reported the mean duration for resolution of mucositis. Cumulative analysis demonstrated that PBM reduced the total duration of OM compared to sham radiation, with a standard mean difference of −1.53 (95% CI −2.14 to −0.92), in favor of the use of PBM (Figure 6) (I^2^ = 0, *p* < 0.05).

### 3.8. Effect on Pain Relief

The impact of PBM on pain relief was reported by three studies [23,24,27]. Gobbo et al. reported the statistically significant improvement of pain on a 0 to 10 numeric pain scale, from 8 on average to 1 in the PBM group and to 2.5 in the control group after 7 days [24]. In another study, the group treated with PBM showed a statistically significant reduction in pain to a score of 0 after 7 days of treatment [23]. However, in the recent study by Legoute et al., there was no difference in pain relief between patients treated with PBM and those receiving sham radiation [27].

### 3.9. Adverse Effects

None of the studies reported any significant adverse events after the use of PBM in the treatment of patients with OM.

### 3.10. GRADE: Summary of Evidence

The trials included in our primary meta-analysis had some concerns of bias due to incomplete outcome data and selective reporting. The results of TSA indicated that the optimal information size was achieved, and the 95% CI excluded the value of no effect. We thereby concluded that the evidence was of moderate quality. The GRADE evidence and summary of findings for our primary outcome are provided in Supplementary Table S3.

## 4. Discussion

Among the very few interventions available for the treatment of oral mucositis due to chemotherapy, PBM has shown significant promise. However, the efficacy and the use of PBM is still a debated topic, in spite of mounting evidence on the use of laser therapy for the treatment of mucositis. PBM is defined as the therapeutic use of light (e.g., visible, near-infrared (NIR), infrared (IR)) absorbed by endogenous chromophores, triggering non-thermal, non-cytotoxic, biological reactions through photochemical or photophysical events, leading to physiological changes [29]. The efficacy of PBM in the treatment of OM in patients undergoing chemotherapy was evaluated in the current study.

The results of this meta-analysis suggest that PBM is effective in reducing the severity of OM, and hence can be recommended for the treatment of OM in patients on cancer chemotherapy. However, I^2^ statistics illustrated a high level of heterogeneity between the studies included in the meta-analysis. After conducting a sensitivity analysis by excluding the studies with high risk of bias, the level of heterogeneity was reduced to 0. The sensitivity analysis illustrated the robustness of the findings from our meta-analysis. Previously, one meta-analysis [14] investigating the effect of PBM as a treatment modality for reducing the severity of OM has suggested that PBM is effective in reducing the severity of OM. However, when a meta-analysis includes only a limited number of RCTs [14], random errors may often lead to deceptive inferences [21]. This emphasizes the need of updating the evidence utilizing recently published trials, taking into account the risks of random errors and gauging the conclusiveness of the current available evidence. Hence, we proceeded with TSA, and the TSA results consolidated the findings of our meta-analysis, suggesting that the evidence provided from the meta-analysis on the benefit of using PBM in reducing the severity of OM is considered conclusive. In pairwise meta-analysis, it is not possible to differentiate whether the meta-analysis is underpowered, and the results can be deemed conclusive [21]. Nevertheless, it is essential to address whether an intervention being investigated is truly effective or not so that it can help researchers as well as policy-makers to assess whether further trials would add informative value or not [30]. Trial sequential analysis can be useful to tackle this concern by differentiating whether the pairwise meta-analyses deliver sufficient evidence for the evaluation of the intervention [21,30].

Subgroup analysis also showed that PBM is effective in the reduction of the duration of OM compared to sham radiation. However, only two studies had reported this outcome. Even though there was no heterogeneity for this analysis, further data would be required for conclusive evidence. Additionally, two studies reported significant reductions in pain after treatment with PBM. A single study reported that PBM was also found to be effective in relieving dysphagia, and improvement of the quality of life [27]. However, data regarding these outcomes were heterogenous and hence not eligible for quantitative analysis.

PBM exhibits anti-inflammatory and growth stimulation properties, making it eligible as an intervention for the treatment of OM. Laser energy is known to be absorbed by chromophores in the respiratory chain, leading to upregulated ATP production, resulting in accelerated tissue repair [31,32,33]. Though the biological mechanisms contributing to the therapeutic benefits of PBM have not been completely understood yet, current evidence from clinical studies indicates that PBM significantly diminishes clinical inflammation and prevents fibrosis [31,32,33]. The effect of LLT on the tissues has been shown to depend on the irradiation parameters, including wavelength density as well as exposure time, cell type and oxidation status [6,34,35,36,37]. There was a notable variation in the laser’s wavelength across the studies, with a wavelength range of 658–970 nm [10]. Only one study by Gobbo et al. utilized a combined wavelength of 660 and 970 nm for the treatment of OM [24]. Other parameters related to PBM, such as the laser’s power, energy density and duration of irradiation, were different in each study. The power of the laser used ranged between 50 and 500 mW, and the energy density ranged from 4 to 6.5 J/cm^2^. Optimal irradiation and dose parameters are likely to vary according to the severity of the underlying pathology, cellular layers affected in the mucosa as well as other patient-associated factors [32,33]. Pathobiological mechanisms of cellular damage manifested as disruptions of tight junctions and matrix metalloproteinase-mediated connective tissue impairment may be a common factor amongst chemotherapeutic regimens. However, different regimens may differ in their cytotoxic potential, which may influence the duration of the resolution of OM. It was not feasible to conduct a quantitative analysis to determine whether these parameters affected the PBM efficacy in OM treatment, due to the limited number of available studies. Further investigation may be required to evaluate the optimal irradiation parameters, as anything lower than the optimum may not have the desired effect, whereas higher doses can have negative impacts. The variation of the laser-related parameters was one of the major limitations in this meta-analysis. Another limitation would be the potential publication bias, which might be attributed to the limited number of RCTs.

There is substantial evidence to affirm the role of PBM in the prevention of OM in patients receiving high-dose chemotherapy conditioning for Bone Marrow Transplantation (BMT) and hematologic malignancies. Hence, PBM was recommended by the MASCC/ISOO in the prevention of OM induced by Hematopoeitic Stem Cell Transplantation (HSCT) conditioned with high-dose CT, with a high level of evidence in adults and adolescents [8]. However, a conclusive recommendation has not been provided regarding PBM for the treatment of OM. A previous meta-analysis has explored the therapeutic efficacy of PBM in reducing the severity of OM and has reported a significant benefit of PBM in the treatment of OM [14]. Our results are in agreement with the aforementioned study and our review presents the most comprehensive summary of findings to date, by the addition of the most recent RCT [27], as well as robust and concrete evidence synthesis by utilizing TSA. Another systematic review in 2013 proposed the use of PBM in the treatment of OM in general, which was supported by the European Society for Medical Oncology (ESMO) clinical practice guidelines for oral mucosal injury [31]. The authors qualitatively reviewed 24 studies on the use of PBM on OM, regardless of the precipitating agent. However, no conclusive guidelines were provided for the prevention or treatment of chemotherapy-induced OM due to a low level of evidence and varying laser parameters. To our knowledge, this is the first attempt to conduct a TSA to evaluate the evidence provided by the meta-analysis, assessing the efficacy of PBM in treating OM.

PBM showed a good safety profile as it was not associated with any serious adverse effects and was reported to be well-tolerated by the patients. Nevertheless, some in vitro studies have found that PBM may trigger oncogenic signaling pathways downstream in tumor cells, increasing the risk of local oral malignancies [38]. However, there has been contradicting evidence as well, indicating the need for more rigorous studies for optimization of laser parameters, especially the density and time. It is also important for the physicians to be mindful to avoid the tumor field during laser application, owing to the risk–benefit ratio. The MASCC/ISOO guidelines recommend informing the patients of this risk, despite the contradicting evidence regarding the risk of developing a malignancy secondary to PBM [39]. Long-term follow-up is also recommended to evaluate the risk of relapse and overall survival of the patient [40]. Additional limitations of PBM include the high price of the laser machines and the need for regular maintenance and personnel training.

## 5. Conclusions

PBM can be considered as an effective agent to treat cancer chemotherapy-induced OM. The results of the current meta-analysis and trial sequential analysis can provide supportive evidence to propose PBM as an acceptable standard of care for the treatment of oral mucositis, with a moderate level of certainty. However, further research with well-designed trials is recommended to investigate the optimal laser settings required for the treatment of OM.

## Figures and Tables

**Table 1:** Population characteristics of studies included in the meta-analysis. RCT: randomized control trial, CT: chemotherapy, RT: radiotherapy, OM: oral mucositis, QoL: quality of life, NCI-CTC AE: National Cancer Institute—Common terminology criteria for adverse events, EORTC: European Organization for research and treatment for cancer scale, WHO: World Health Organization, Sham: placebo radiation.

Author	Intervention	Control	PBM Duration	Irradiation Time	Primary Outcome	OM Assessment	Secondary Outcomes	Other Outcomes
Kuhn 2007 [25]	Laser 830 nm, 100 mW	Sham	5 days	N/A	Reduction in severity (OM grade)	NCI-CTCAE	OM duration	Nil
Genot-Klastersky 2008 [28]	Visible laser + Infrared laser 100–500 mW	Sham	3 days	6 min	Reduction in severity (OM grade)	EORTC	OM progression	Esophageal OM
Kuhn 2009 [26]	Laser 830 nm, 100 mW	Sham	5 days	N/A	Reduction in severity (OM grade)	NCI-CTCAE v2	OM duration	Nil
Amadori 2016 [23]	Laser 830 nm, 150 mW	Sham	4 days	30 s	Reduction in severity (OM grade)	WHO	Pain score (VAS)	Nil
Gobbo 2018 [24]	Laser 970 nm, 250 mW	Sham	4 days	25 s	Reduction in severity (OM grade)	WHO	OM grade on day 4 and 11	Pain/analgesic use
Legoute 2019 [27]	Laser 658 nm, 100 mW	Sham	5 days	40 s	Reduction in severity (OM grade)	WHO	Pain/consumption of analgesics	Nutritional status, compliance to cancer treatment, QoL

## Data Availability

Not applicable.

## Supplementary Materials

The following are available online at https://www.mdpi.com/article/10.3390/ijerph18147418/s1, Figure S1: Funnel plot; Table S1 and S2: Details of the search strategy and excluded articles; Table S3: Grade summary of findings.
