# Supplementary material for: Efficacy of Photobiomodulation in the Treatment of Cancer Chemotherapy-Induced Oral Mucositis: A Meta-Analysis with Trial Sequential Analysis
Source: Int J Environ Res Public Health. 2021 Jul 12;18(14):7418. doi: 10.3390/ijerph18147418 (PMC8307997; doi:10.3390/ijerph18147418)
Supplement: Supplementary file 1 [file ijerph-18-07418-s001.zip › ijerph-1250552-supplementary.pdf]

Figure S1: Funnel plot illustrating publication bias for the included studies.

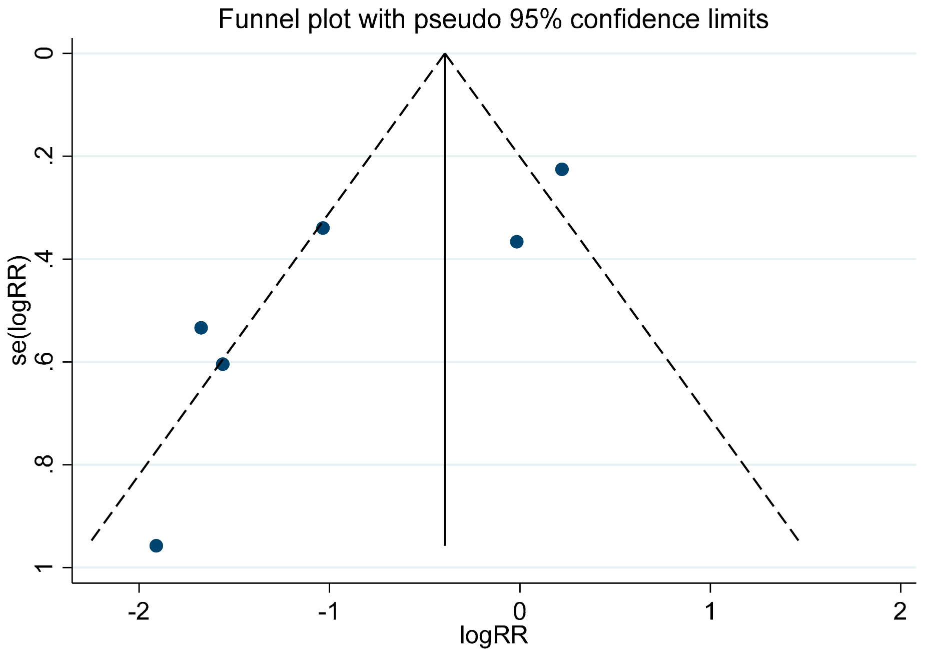

Table S1: (Low level laser therapy) search strategy and results on 07/06/2020

#	**Keyword**	Medline	Embase	CENTRAL
1	exp NEOPLASMS/	3322696	4875367	77595
2	exp LEUKEMIA/	230863	335868	4644
3	exp LYMPHOMA/	170770	324641	3173
4	exp RADIOTHERAPY/	184310	598222	5856
5	exp Antineoplastic agents/	1091501	2344609	54485
6	Bone Marrow Transplantation/	44495	53048	1368
7	neoplasm$.mp.	2776478	874920	79397
8	cancer$.mp.	1535543	3712965	169046
9	(leukaemi$ or leukemi$).mp.	307609	503434	15026
10	(tumour$ or tumor$).mp.	1901438	3381384	78405
11	malignan$.mp.	503027	953635	27680
12	neutropeni$.mp.	40925	130357	14301
13	carcino$.mp.	940747	1627071	45899
14	adenocarcinoma$.mp.	220135	293463	11190
15	lymphoma$.mp.	225844	343706	12414
16	(radioth$ or radiat$ or irradiat$).mp.	787768	1446902	51221
17	(bone adj marrow adj5 transplant$).mp.	56629	90028	4001
18	chemo$.mp.	673250	1369673	92912
19	or/1-18	5188139	8427225	292944
20	exp STOMATITIS/	16657	53767	1024
21	Candidiasis, Oral/	4675	3890	213
22	stomatitis.mp.	23286	46240	3995
23	mucositis.mp.	8904	18284	3488
24	(oral adj6 mucos$).mp.	20290	34044	3078
25	(mycosis or mycotic).mp.	16655	81913	1489
26	mIAS.ti,ab.	313	544	20
27	or/20-26	71791	188196	9678
28	low level laser therapy	5681	23423	2
29	Phototherapy/ or Phototherap$	13743	29506	3151
30	Laser Biostimulation.mp.	63	115	20
31	Light Therapy	7313	3584	1908
32	Laser therapy	42073	28903	5184
33	Photobiomodulation	814	1605	452
34	LLLT.mp.	1502	2449	812
35	Laser/	36129	83153	593
36	laser Irradiation	6750	9671	677
37	Low level/ or Low-level.ti,ab.	53394	79725	3810
38	Low power/ or Low-power.ti,ab.	5225	9560	550
39	OR/28-37	147744	223227	12645
40	random$.ti,ab.	955662	1553420	956392

41	placebo$.ti,ab.	193683	314382	298344
42	(doubl$ adj blind$).ti,ab.	136488	215598	239421
43	(singl$ adj blind$).ti,ab.	15777	25139	27309
44	allocat$.ti,ab.	98906	154053	75281
45	clinical study/ or clinical trial/ or controlled clinical trial/	548728	1217093	53
46	OR/39-44	1438705	2616621	1068705
47	19 AND 27 AND 38 AND 45	150	396	143

Table S2 List of excluded articles after full-text review with reason

#	Author/Year	Title	Reason
1	Bensadoun 1999	Low-energy He/Ne laser in the prevention of radiation-induced mucositis: A multicenter phase III randomized study in patients with head and neck cancer	Prophylaxis
2	Toida M 2003	Usefulness of Low-Level Laser for Control of Painful Stomatitis in Patients with Hand-Foot-and-Mouth Disease	Non-oncology related stomatitis
3	Antunes 2007	Low-power laser in the prevention of induced oral mucositis in bone marrow transplantation patients: A randomized trial	Prophylaxis
4	Schubert 2007	A phase III randomized double-blind placebo-controlled clinical trial to determine the efficacy of low level laser therapy for the prevention of oral mucositis in patients undergoing hematopoietic cell transplantation	Prophylaxis
5	Nasrin 2009	Relieving pain in minor aphthous stomatitis by a single session of non-thermal carbon dioxide laser irradiation.	Non-oncology related stomatitis
6	Adnan 2009	An evaluation of different treatments for recurrent aphthous stomatitis and patient perceptions: nd: YAG laser versus medication	Non-oncology related stomatitis
7	Šimunović-Šoškić 2010	Salivary Levels of TNF-α and IL-6 in Patients with Denture Stomatitis Before and After Laser Phototherapy	Irrelevant outcome
8	Clarkson 2010	Interventions for treating oral mucositis for patients with cancer receiving treatment	Non-RCT
9	Ana 2010	Low-power laser to prevent oral mucositis in autologous hematopoietic stem cell transplantation.	Prophylaxis
10	Silva 2011	The prevention of induced oral mucositis with low-level laser therapy in bone marrow transplantation patients: a randomized clinical trial	Prophylaxis
11	Sharon 2011	A randomized controlled trial of visible-light therapy for the prevention of oral mucositis	Prophylaxis
12	Gautam 2012	Low level laser therapy for concurrent chemoradiotherapy induced oral mucositis in head and neck cancer patients - a triple blinded randomized controlled trial.	Prophylaxis
13	Angelica Ferreira 2012	Effect of intraoral low-level laser therapy on quality of life of patients with head and neck cancer undergoing radiotherapy.	Irrelevant outcome
14	Gautam AP 2012	Low level helium neon laser therapy for chemoradiotherapy induced oral mucositis in oral cancer patients - a randomized controlled trial.	Prophylactic (Duplicate with 14)
15	P.U. Prakash Saxena 2012	Effects of low-level laser therapy in the prevention and treatment of concurrent chemoradiotherapy induced oral mucositis: A triple-blind randomized controlled trial.	Prophylactic
16	Gautam AP 2012	Low level laser therapy for concurrent chemoradiotherapy induced oral mucositis in head and	Prophylactic Duplicate with 12

		neck cancer patients - A triple blinded randomized controlled trial	
17	Hodgson 2012	Amelioration of oral mucositis pain by NASA near-infrared light-emitting diodes in bone marrow transplant patients	Prophylactic
18	Zand 2012	Promoting wound healing in minor recurrent aphthous stomatitis by non-thermal, non-ablative CO(2) laser therapy: a pilot study	Non-oncology related stomatitis
19	Gouvea de Lima A de 2012	Oral mucositis prevention by low-level laser therapy in head-and-neck cancer patients undergoing concurrent chemoradiotherapy: a phase III randomized study.	Prophylaxis
20	Sattayut 2013	A clinical efficacy of using CO2 laser irradiating to transparent gel on aphthous stomatitis patients	Non-oncology related stomatitis
21	Arbabi-Kalati 2013	Evaluation of the effect of low level laser on prevention of chemotherapy-induced mucositis	Prophylaxis
22	Oton-Leite 2013	Effect of low level laser therapy in the reduction of oral complications in patients with cancer of the head and neck submitted to radiotherapy	Prophylaxis
23	Prasad 2013	Assessment of immediate pain relief with laser treatment in recurrent aphthous stomatitis	Non-oncology related stomatitis
24	Antunes 2013	Phase III trial of low-level laser therapy to prevent oral mucositis in head and neck cancer patients treated with concurrent chemoradiation	Prophylaxis
25	Gautam 2013	Effect of low-level laser therapy on patient reported measures of oral mucositis and quality of life in head and neck cancer patients receiving chemoradiotherapy--a randomized controlled trial.	Prophylactic
26	Albrektson, Margit 2014	Recurrent aphthous stomatitis and pain management with low-level laser therapy: a randomized controlled trial	Non-oncology related stomatitis
27	de Paula Eduardo 2015	Efficacy of cryotherapy associated with laser therapy for decreasing severity of melphalan-induced oral mucositis during hematological stem-cell transplantation: a prospective clinical study	Prophylaxis
28	Silva GB 2015	Effect of low-level laser therapy on inflammatory mediator release during chemotherapy-induced oral mucositis: a randomized preliminary study	Prophylaxis
29	Angelica F. Oton-Leite 2015	Effect of low-level laser therapy on chemoradiotherapy-induced oral mucositis and salivary inflammatory mediators in head and neck cancer patients	Prophylaxis
30	A.0P., Gautam 2015	Low level laser therapy against radiation induced oral mucositis in elderly head and neck cancer patients-a randomized placebo controlled trial	Prophylaxis
31	C., Antoniou 2016	A multicenter, randomized, split-face clinical trial evaluating the efficacy and safety of chromophore gel-assisted blue light phototherapy for the treatment of acne	Non-oncology related stomatitis
32	C.M., Maciel 2016	Methylene Blue-Mediated Photodynamic Inactivation Followed by Low-Laser Therapy versus Miconazole Gel in the Treatment of Denture Stomatitis	Non-oncology related stomatitis

33	Antunes HS 2016	Cost-effectiveness of low-level laser therapy (LLLT) in head and neck cancer patients receiving concurrent chemoradiation	Irrelevant outcome
34	Ferreira, Betania 2016	Low-level laser therapy prevents severe oral mucositis in patients submitted to hematopoietic stem cell transplantation: a randomized clinical trial	Prophylaxis
35	J.K.H., Ho 2017	Prevention and treatment of oral mucositis caused by chemo and radiotherapy in head and neck cancer patient	Non-RCT
36	Salvador DRN 2017	Effect of photobiomodulation therapy on reducing the chemo-induced oral mucositis severity and on salivary levels of CXCL8/interleukin 8, nitrite, and myeloperoxidase in patients undergoing hematopoietic stem cell transplantation: a randomized clinical trial	Prophylaxis
37	H.G., Yilmaz 2017	Treatment of recurrent aphthous stomatitis with Er,Cr:YSGG laser irradiation: A randomized controlled split mouth clinical study	Prophylaxis
38	Antunes HS 2017	Long-term survival of a randomized phase III trial of head and neck cancer patients receiving concurrent chemoradiation therapy with or without low-level laser therapy (LLLT) to prevent oral mucositis	Prophylaxis
39	Rozza-de-Menezes 2018	Behaviour and Prevention of 5'Fluorouracil and Doxorubicin-induced Oral Mucositis in Immunocompetent Patients with Solid Tumors: A Randomised Trial	Prophylaxis
40	Queiroz 2018	Arrabidaea chica for oral mucositis in patients with head and neck cancer: A protocol of a randomised clinical trial	Non-RCT (RCT protocol)
41	Antunes 2018	cDNA microarray analysis of human keratinocytes cells of patients submitted to chemoradiotherapy and oral photobiomodulation therapy: pilot study	Irrelevant outcome
42	Martins 2019	Effect of photobiomodulation on the severity of oral mucositis and molecular changes in head and neck cancer patients undergoing radiotherapy: A study protocol for a cost-effectiveness randomized clinical trial	Irrelevant outcome
43	Marin-Conde 2019	Photobiomodulation with low-level laser therapy reduces oral mucositis caused by head and neck radio-chemotherapy: prospective randomized controlled trial	Prophylaxis
44	Cruz 2007	Influence of low-energy laser in the prevention of oral mucositis in children with cancer receiving chemotherapy	Prophylaxis
45	Medeiros-Filho 2017	Laser and photochemotherapy for the treatment of oral mucositis in young patients: Randomized clinical trial.	Both groups received phototherapy (Co-intervention)
46	Gobbo 2017	Multicenter randomized double-blind controlled trial to evaluate the efficacy of laser therapy for treatment of severe oral mucositis induced by chemotherapy in children. Lampo RCT	Identical to 5 in inclusion list (earlier version)

47	Vitale 2017	Preliminary study in a new protocol for the treatment of oral mucositis in pediatric patients undergoing hematopoietic stem cell transplantation (HSCT) and chemotherapy (CT)	Non-RCT
48	Soares 2018	Treatment of mucositis with combined 660- and 808-nm-wavelength low-level laser therapy reduced mucositis grade, pain, and use of analgesics: a parallel, single-blind, two-arm controlled study	Both groups received phototherapy (Infra-red vs LLLT)
49	Ribeiro da Silva 2018	Photodynamic therapy for treatment of oral mucositis: Pilot study with pediatric patients undergoing chemotherapy	both groups received phototherapy

Table S3: GRADE summary of findings

Summary of findings:

Low-Level Laser Therapy compared to SHAM for Treatment of oral mucositis

Patient or population: [health problem and/or population]

Setting:

Intervention: [intervention]

Comparison: [comparison]

Outcomes	**Anticipated absolute effects*** (95% CI)		Relative effect (95% CI)	№ of participants (studies)	Certainty of the evidence (GRADE)	Comments
	**Risk with [comparison]**	**Risk with [intervention]**				
Reduction in severity	424 per 1,000	**182 per 1,000** (85 to 395)	**RR 0.43** (0.20 to 0.93)	398 (6 RCTs)	⨁⨁⨁◯ MODERATE	

***The risk in the intervention group** (and its 95% confidence interval) is based on the assumed risk in the comparison group and the **relative effect** of the intervention (and its 95% CI).

CI: Confidence interval; **RR:** Risk ratio

GRADE Working Group grades of evidence
High certainty: We are very confident that the true effect lies close to that of the estimate of the effect
Moderate certainty: We are moderately confident in the effect estimate: The true effect is likely to be close to the estimate of the effect, but there is a possibility that it is substantially different
Low certainty: Our confidence in the effect estimate is limited: The true effect may be substantially different from the estimate of the effect
Very low certainty: We have very little confidence in the effect estimate: The true effect is likely to be substantially different from the estimate of effect
